# MetaComBin: combining abundances and overlaps for binning metagenomics reads

**DOI:** 10.3389/fbinf.2025.1504728

**Published:** 2025-03-03

**Authors:** Francesco Tomasella, Cinzia Pizzi

**Affiliations:** Department of Information Engineering, University of Padova, Padua, Italy

**Keywords:** metagenomics, reads binning, abundance, overlap, k-mers, clustering

## Abstract

**Introduction:**

Metagenomics is the discipline that studies heterogeneous microbial samples extracted directly from their natural environment, for example, from soil, water, or the human body. The detection and quantification of species that populate microbial communities have been the subject of many recent studies based on classification and clustering, motivated by being the first step in more complex pipelines (e.g., for functional analysis, de novo assembly, or comparison of metagenomes). Metagenomics has an impact on both environmental studies and precision medicine; thus, it is crucial to improve the quality of species identification through computational tools.

**Methods:**

In this paper, we explore the idea of improving the overall quality of metagenomics binning at the read level by proposing a computational framework that sequentially combines two complementary read-binning approaches: one based on species abundance determination and another one relying on read overlap in order to cluster reads together. We called this approach MetaComBin (metagenomics combined binning).

**Results and Discussion:**

The results of our experiments with the MetaComBin approach showed that the combination of two tools, based on different approaches, can improve the clustering quality in realistic conditions where the number of species is not known beforehand.

## 1 Introduction

Microbes influence everyday life in countless ways, from helping modulate the atmosphere to keeping animals (including humans) and plants in a healthy status and helping detect environmental pollution and disease spread. Traditional genomic-based approaches require prior clone and laboratory culturing for further investigation (Felczykowska et al., 2012). However, not all bacteria can be cultured in a laboratory, as they might require habitat conditions that cannot easily be reproduced. Moreover, in a laboratory culture, the presence of multiple species, which is the norm in living environments, is usually considered contamination, thus preventing holistic study.

The advent of metagenomics has revolutionized the field of microbiology by shifting the focus from the individual microbe study to that of a complex microbial community. Metagenomics is the study of heterogeneous microbial communities by directly sampling the natural environment in which they live and sequencing the entire microbial community it contains (Kang et al., 2015). Samples can be taken from a variety of environments (e.g., soil, water, or the human gut or saliva, etc.) with the primary goal of determining the taxonomic identity of the microorganisms that are present in the samples (Staley and Konopka, 1985). Microbial studies play a prominent role in both environmental studies and precision medicine. In fact, among the advantages of metagenomics is the possibility of studying interactions among microbes living in the same environment (Shreiner et al., 2015) and comparing samples taken from similar environments or at different points in time for environmental monitoring or health screenings (e.g., Ondov et al., 2016; Pellegrina et al., 2020).

Alongside opening new research perspectives, metagenomics brings both experimental challenges for correct environmental sampling and computational challenges for quality control, assembly, and taxonomic and functional classification of large-scale complex communities (Bharti and Grimm, 2021). In particular, the detection and quantification of the species in a metagenomics sample is of paramount interest both as a challenging computational problem *per se* and as the first step in complex pipelines for functional analysis and sample comparisons (Mande et al., 2012). Despite extensive studies, accurate identification at the read level remains challenging (Sczyrba et al., 2017; Comin et al., 2021). Supervised methods can obtain high precision levels, but they rely on reference database completeness. Moreover, the construction of a k -mers DB usually requires computing capabilities with large amounts of RAM and disk space. Another drawback is the inherent incompleteness of available databases: most bacteria found in environmental samples are unknown and cannot be cultured and separated in the laboratory; thus, their genome is not yet present in reference databases. For these reasons, the number of unassigned reads can be very high when using supervised methods (Lindgreen et al., 2013; Girotto et al., 2017a).

On the other hand, unsupervised classification tools, also known as metagenome binning algorithms, are based on the observation that the k -mer distributions of the DNA fragments from the same genome are more similar than those from different genomes. Thus, without using any reference genome, one can determine if two fragments are from genomes of similar species based on their k -mer distributions. In this study, we will focus on the unsupervised detection of species in a sample without the use of reference genomes and consider the short reads provided by the sequencing process as fragments.

One of the major problems when processing metagenomic data is the fact that the proportion of species in a sample, that is, the abundance rate, can vary greatly. Some tools, for example, AbundanceBin (Wu and Yuzhen, 2011), explicitly exploit this variability, clustering reads based on their abundance ratio. Although this approach works well if all the species in the sample have a different abundance, the approach struggles to distinguish among species with the same abundance. Approaches based on exploiting read overlaps and subsequently clustering them are capable of better distinguishing among single species, even if their abundance is similar. In recent years several such approaches have been proposed, mainly differing in the techniques used for feature extraction and the distance measure they use to define similarity (Wang et al., 2012; Vinh et al., 2015; Girotto et al., 2016; Andreace et al., 2021; Balvert et al., 2021).

In this paper^1^, we explore the idea of improving the overall quality of metagenomics binning at the read level by proposing a metagenomic combined binning framework, MetaComBin, that sequentially combines two complementary read-binning approaches. Read binning is intrinsically more difficult than contig binning, especially when short reads are used due to the limited length that can be exploited to compute statistics on the read itself. The authors of AbundanceBin tried a similar approach, combining their tool with MetaCluster. However, their experiments assumed that the exact number of species in the sample was known beforehand. Motivated by the curiosity of testing this idea on a more realistic framework, we paired AbundanceBin with MetaProb, which has the capability of estimating the number of species and proved in separate experiments Girotto et al. (2016) to outperform MetaCluster.

We run experiments on three datasets with several species, some of which, but not all, occur in the sample with the same abundance. The datasets we chose were among the most difficult to cluster by state-of-the-art methods for read binning, according to previous studies (Girotto et al., 2016). Our experiments suggest that our intuition is correct and that the combination of complementary tools can indeed be beneficial for metagenomic binning at the read level in more realistic, unsupervised settings.

## 2 Materials and methods

In this section, we will describe MetaComBin, the combined framework we used for our analysis, starting with the methodological details of the two tools we used: AbundanceBin (Wu and Yuzhen, 2011) and MetaProb (Girotto et al., 2016).

### 2.1 AbundanceBin

AbundanceBin is a tool for metagenomic binning based on abundance estimation. One of its strengths is its ability to produce satisfactory binning results even when the reads are very short (approximately 75 bp). The tool can work in an unsupervised manner, not requiring any information regarding the number of bins, similar to the data obtained in real and non-simulated situations when we do not know the composition of the samples. The working hypothesis of AbundanceBin is that the distribution of reads follows the Lander–Waterman model, whereby the coverage of the various nucleotide positions is modeled via a Poisson distribution. The metagenomics sequencing procedure can be viewed as a set of Poisson distributions, each of which represents a different species. In the presence of *m* different species, therefore, it is possible to identify *m* Poisson distributions. The mean of each of these distributions represents the abundance of the species and is, therefore, the element that must be calculated to obtain an estimate of their abundance.

AbundanceBin thus solves an optimization problem using an expectation-maximization (EM) algorithm. Once the EM algorithm has converged, it is possible to calculate the probability of assigning a read to a bin, even if there is the possibility that the read remains unassigned. The EM algorithm requires the number of bins as input. To solve this problem, AbundanceBin adopts a recursive approach that is based on dividing the dataset into two bins, subsequently iterating the process until bins with very different abundances are obtained. AbundanceBin performs well in situations in which the abundance of species is different, although not less than a 1:2 ratio. In cases with less variability when the species have a comparable abundance, AbundanceBin is no longer an optimal choice and shows very high error rates because it will most likely group different species with a similar abundance in the same bin.

### 2.2 MetaProb

MetaProb is a two-step approach for metagenomic read binning. The reads are first grouped together based on their overlap, measured in terms of the number of shared q -mers, with q=31 by default. The output of this phase is a relatively large number of small groups of very connected reads that are, therefore, likely to belong to the same species. Next, within each group, a set of representative, not overlapping (to avoid redundancy) reads is chosen, and from it, an l -mer profile ( l=5 by default) is extracted and normalized to obtain a group signature. Such signatures are finally given in input to the k -means clustering algorithm that will group signatures (and their corresponding groups of reads) to obtain the final clusters that represent the different species in the sample.

Similarly to EM, the k -means algorithm also requires previous knowledge of the number k of clusters to obtain. MetaProb can both accept this parameter as input or estimate the value of k by exploiting the Kolmogorov–Smirnov test.

### 2.3 Combined framework: MetaComBin

The idea of our framework is a two-step approach. First, in Step 1, we partition the reads so that all the reads of species with the same abundance are clustered with the AbundanceBin abundance-based algorithm.

**FIGURE 1:**
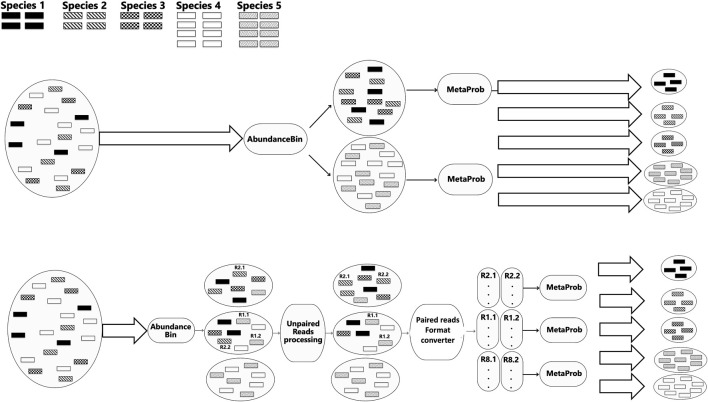
Ideal pipeline of the MetaComBin combined framework (top figure) and a more realistic pipeline (bottom figure).

Next, in Step 2, the MetaProb overlap-based approach is applied to each of the obtained clusters in order to separate the species within it. Figure 1 (top) shows the ideal pipeline of our approach. In reality, especially if the final number of expected species is not known and given, the combination of the two tools is not as smooth as in the ideal pipeline. In addition to the fact that none of the currently available read-binning algorithms is capable of perfect clustering, AbundanceBin, unlike most read-binning tools (including MetaProb), has not been designed to take into account paired-end reads. This means that it is possible that reads that are paired (and thus belong to the same species) are assigned by AbundanceBin to different clusters. This raises the problem of how to deal with paired-end reads that have been wrongly separated by AbundanceBin. In addition to being a conceptual error from AbundanceBin, this also creates a practical problem at the following step because MetaProb needs sets of paired-end reads as input. Possible options include deleting all unpaired reads or designing a reassignment strategy. However, determining the destination cluster for each read is complex, requiring a case-by-case evaluation based on the obtained results and the overall composition of the clusters. In our experiments, we explored two possible approaches: i) the reassignment of unpaired reads only from clusters with a very high percentage of unpaired reads; ii) the reassignment of unpaired reads starting from the cluster with the highest percentage of unpaired reads, and iteration of the reassignment until no more unpaired reads remain.

 More formally, let $D=\left\{r_{ij}|i=1,\ldots n,\;j=1,2\right\}$ be the input dataset of paired-end reads, and let $\left(r_{i1},r_{i2}\right)$ be the paired-end couple i , |D|=2n . Let $C=\left\{C_{1},C_{2},\ldots ,C_{m}\right\}$ be the set of clusters obtained by AbundanceBin. Because AbundanceBin considers each read separately, it is possible for a pair $p\;\left(r_{p1},r_{p2}\right)$ that $r_{p1}\in C_{h}$ and $r_{p2}\in C_{k}$ with h≠k . Let $U_{i}$ be the subset of unpaired reads in $C_{i}$ . The two approaches we tested are:

1. Static reassignment: given a threshold T . For each cluster $C_{i}$ with $|U_{i}|/|C_{i}|\geq T$ , move each read in $U_{i}$ in the cluster of its paired read; that is, if $r_{p1}\in C_{i}$ and $r_{p2}\in C_{t}$ , move $r_{p1}$ in $C_{t}$ .
2. Iterative reassignment: sort the clusters in C by decreasing number of unpaired reads. Starting from $C_{1}$ , the cluster with the highest number of unpaired reads, reassign its unpaired reads in $U_{1}$ to the clusters where their paired read is. Iterate the process by considering $C_{2}$ and continue until there are no more unpaired reads.

In Figure 1 (bottom), we can see a more realistic pipeline with the added intermediate processing.

## 3 Results and discussion

In this section, we will describe the dataset composition and the clustering quality measures used to assess the methods. Then, we will discuss the results obtained for the analysis of each of the considered datasets. The two tools, AbundanceBin and MetaProb, were run with default parameters. We did all the experiments *without* giving the number of expected clusters in input to mimic a more realistic context. All the experiments were performed on a machine with Intel(R) Xeon(R) Gold 5220 CPUs @ 2.20/3.90 GHz and 2TB of RAM.

### 3.1 Datasets

For our analysis, we chose three datasets used in several previous papers on metagenomic read binning (Girotto et al., 2017a; Vinh et al., 2015; Girotto et al., 2016; Andreace et al., 2021). We chose the datasets S7, S9, and MIXK, which are suitable for verifying our hypothesis, that is, whether using multiple clustering phases leads to improved results compared to using a single read-binning tool.

The datasets S7 and S9 were produced by Vinh et al. (2015), and they were downloaded by MetaProb repository^2^. The S7 dataset is composed of short paired-end reads and includes five species with abundance ratios 1:1:1:4:4 and with phylogenetic distance at the order and genus levels. The dataset was simulated using MetaSim, a tool for generating metagenomic reads, using the Illumina error profile with an error rate of 1%. Details of the dataset composition are given in Table 1.

**TABLE 1:** Details of the dataset S7.

Species	Name	Coverage	Paired reads
Species 1	*Actinobacillus pleuropneumoniae serovar* 5b str. L20	10	141,928
Species 2	*Aliivibrio salmonicida* LFI1238	10	75,183
Species 3	*Haemophilus* *somnus* 129PT	10	126,183
Species 4	*Pasteurella* *multocida* 36950	40	588,088
Species 5	*Vibrio cholerae* M66-2	40	722,168

The S9 dataset was also simulated using MetaSim and obtained according to the same error profile. However, it was reduced in size with respect to the original file because AbundanceBin was not capable of analyzing it. From the original S9 dataset, we selected 10 species with abundance ratios 1:1:2:2:2:2:3:3:3:3. Their phylogenetic distance was at either the phylum or the family level. We called this dataset S9red. Details of the dataset composition are given in Table 2.

The MIXK dataset was derived from a synthetic dataset originally produced by the authors of the popular Kraken metagenomic classifier (Wood and Salzberg, 2014). The reads in this dataset were not simulated but were obtained by combining real sequences obtained from projects that sequenced isolated microbial genomes. When creating these synthetic metagenomes, they used data sequenced by the Illumina HiSeq sequencing platform, which is available either at the GAGE-B project or on the NCBI Sequence Read Archive (details are on the Kraken paper). The MIXK dataset contains the reads of the species listed in Table 3 with an abundance ratio of 1:1:1:1:1:1:2:2:3:6.

**TABLE 2:** Details of the reduced dataset S9red.

Species	Name	Coverage	Paired reads
Species 1	*Ehrlichia* *canis* str. Jake	10	82,012
Species 2	*Desulfovibrio vulgaris* DP4	10	216,912
Species 3	*Bartonella clarridgeiae* 73 5	5	47,629
Species 4	*Caldicellulosiruptor lactoaceticus* 6A	10	167,267
Species 5	*Lactobacillus* *amylovorus* GRL1118	15	177,691
Species 6	*Streptococcus* *thermophilus* JIM 8232	15	181,562
Species 7	*Helicobacter* *cetorum* MIT 00-7128	15	182,576
Species 8	*Bifidobacterium animalis* subsp. lactis B420	5	60,623
Species 9	*Mesotoga prima* MesG1.Ag.4.2	15	279,117
Species 10	*Geobacter sulfurreducens* PCA	10	238,138

**TABLE 3:** Details of synthetic dataset MIXK.

Species	Name	Coverage	Paired reads
Species 1	*Streptococcus* *pneumoniae* TIGR4	20	91,532
Species 2	*Xanthomonas axonopodis* pv. Manihotis UA323	60	308,021
Species 3	*Bacillus* *cereus* VD118	10	59,775
Species 4	*Aeromonas* *hydrophila* SSU	10	51,766
Species 5	*Mycobacterium* *bscessus* 6G-0125-R	10	54,252
Species 6	*Rhodobacter sphaeroides* 2.4.1	20	97,723
Species 7	*Pelosinus fermentans* A11	10	52,623
Species 8	*Bacteroides fragilis* HMW	10	56,146
Species 9	*Vibrio cholerae* CP1032(5)	30	167,707
Species 10	*Staphylococcus aureus* M0927	10	60,455

### 3.2 Evaluation metrics

To evaluate the quality of the results of the binning tools involved in this study, we used four popular performance evaluation metrics (namely, precision, recall, F-measure and ARI) as defined in other read-binning papers (Girotto et al., 2017a; Vinh et al., 2015; Girotto et al., 2016; Andreace et al., 2021; Balvert et al., 2021; Girotto et al., 2017b) and displayed in Equations 1–4. Let n be the number of species in the simulated dataset, and let C be the number of clusters returned by the algorithm. $A_{ij}$ is the number of reads from species j assigned to cluster i , $A_{i}$ is the sum over all j of the values $A_{ij}$ for each given i , and similarly $A_{j}$ is the sum over all i of the values $A_{ij}$ for each given j :

$$Precision=\frac{\sum_{i=1}^{C}\max_{j}A_{ij}}{\sum_{i=1}^{C}\sum_{j=1}^{n}A_{ij}},$$ (1)

$$Recall=\frac{\sum_{j=1}^{n}\max_{i}A_{ij}}{\sum_{i=1}^{C}\sum_{j=1}^{n}A_{ij}+\text{\#unassigned reads}},$$ (2)

$$\text{F}-\text{measure}=\frac{2*Precision*Recall}{Precision+Recall},$$ (3)

$$ARI=\frac{\sum_{ij}\left({­}_{2}^{{\;A}_{ij}}\right)-\left[\sum_{i}\left({­}_{2}^{{\;A}_{i}}\right)\times \sum_{j}\left({­}_{2}^{{\;A}_{j}}\right)\right]/\left({­}_{2}^{n}\right)}{0.5\times \left[\sum_{i}\left({­}_{2}^{{\;A}_{i}}\right)+\sum_{j}\left({­}_{2}^{{\;A}_{j}}\right)\right]-\left[\sum_{i}\left({­}_{2}^{{\;A}_{i}}\right)\times \sum_{j}\left({­}_{2}^{{\;A}_{j}}\right)\right]/\left({­}_{2}^{n}\right)}.$$ (4)

### 3.3 Experimental analysis of dataset S7

#### 3.3.1 Step 1: clustering of reads based on abundances

We obtained three different clusters from AbundanceBin, one more than expected. From Table 4, we can see the actual partition, and, in particular, we can notice the presence of paired reads that have been assigned to different clusters and their abundance in the cluster. The three resulting clusters highlight an effective grouping of the species with the highest abundance, that is, Species 4 and 5 (as illustrated in Table 5), which are mainly located within Cluster 1. The three species with lower abundance are instead distributed between Cluster 2 and Cluster 3, the latter mainly characterized by the presence of unpaired reads.

**TABLE 4:** Clusters compositions generated by AbundanceBin on the dataset S7.

Cluster	Total reads	Paired reads	Unpaired reads	% Unpaired reads
Cluster 1	2,405,443	2,152,948	252,495	10.5%
Cluster 2	786,613	453,664	332,949	42.33%
Cluster 3	115,044	20,506	94,538	82.18%

**TABLE 5:** Number of reads per species in the clusters generated by AbundaceBin on the dataset S7.

Cluster	Species 1	Species 2	Species 3	Species 4	Species 5
Cluster 1	10,000	18,723	8,097	1,065,139	1,303,484
Cluster 2	225,827	108,303	203,762	109,648	139,073
Cluster 3	48,029	23,340	40,507	1,389	1,779

Although the obtained number of clusters differs from the actual one, when looking in more detail at the composition of the clusters, we can state that the partitioning of the species within the clusters is congruent with the expected abundance, demonstrating the effectiveness of AbundanceBin in identifying and grouping species with the same abundance in dataset S7.

Tables 6–8 highlight how many of the associated reads are actually paired or unpaired for each species within each cluster. The % Read column also displays the structure of the cluster and the percentages of the constituent species.

**TABLE 6:** Detailed composition of Cluster 1 obtained from AbundanceBin on S7.

Species	Reads	% Reads	Paired	Unpaired
Species 1	10,000	0.42%	30.04%	69.96%
Species 2	18,723	0.78%	27.98%	72.02%
Species 3	8,097	0.34%	30.04%	69.96%
Species 4	1,065,139	44.30%	90.62%	9.38%
Species 5	1,303,484	54.16%	90.30%	9.70%

**TABLE 7:** Detailed composition of Cluster 2 obtained from AbundanceBin on S7.

Species	Reads	% Reads	Paired	Unpaired
Species 1	225,827	28.70%	80.51%	19.49%
Species 2	108,303	13.77%	74.09%	25.91%
Species 3	203,762	25.90%	81.82%	18.18%
Species 4	109,648	13.94%	9.90%	90.10%
Species 5	139,073	17.69%	10.10%	89.90%

**TABLE 8:** Detailed composition of Cluster 3 obtained from AbundanceBin on S7.

Species	Reads	% Reads	Paired	Unpaired
Species 1	48,029	41.75%	18.46%	81.54%
Species 2	23,340	20.29%	17.93%	82.07%
Species 3	40,507	35.21%	18.40%	81.60%
Species 4	1,389	1.21%	0%	100%
Species 5	1,779	1.55%	0%	100%

As can be seen, more than 90% of the reads of the species with the highest abundance are included in Cluster 1. The remaining 10%, mainly composed of unpaired reads, are distributed between Clusters 2 and Cluster 3. The reads of the species with lower abundance are mainly distributed between Cluster 2 and Cluster 3, with some smaller quantities erroneously assigned to Cluster 1. For each of these three species, it is noteworthy that the vast majority of reads are assigned to Cluster 2 as a pair of paired-end reads, while the opposite trend is observed within Cluster 3, in which the prevalence of reads for each species consists of unpaired reads.

The computed values of precision, recall, and F-measure for AbundanceBin are 0.47, 0.87, and 0.61, respectively, in line with those obtained by Girotto et al. (2016). The low value of precision is expected because the AbundanceBin principle is to cluster together reads with the same abundance that, in our case study, can belong to different species. Similarly, we expect a high recall value because most reads of the same species will be included in the same cluster.

#### 3.3.2 Step 2: partitioning of the abundance clusters

Before applying MetaProb to each of the clusters obtained with AbundanceBin, processing was needed to avoid the presence of unpaired reads. We tried two approaches:

1. Reassign all unpaired reads of clusters with a composition of unpaired reads above a threshold T (we chose T=80% ) to the cluster of their paired read;
2. Reassign all unpaired reads of the cluster with the highest percentage of unpaired reads; iterate the process until no more unpaired reads are left.

In practice, in our case study, with Approach 1, we simply reassigned the unpaired reads of Cluster 3 to their counterparts in Cluster 1 and Cluster 2 and then removed any other unpaired reads that were left. With Approach 2, we did not remove any reads, which resulted in more than 250,000 reads re-paired with respect to Approach 1. We report here in detail only the results of the iterative Approach 2 because it appears to be the most effective one, as we will discuss later. The details of this processing are shown in Tables 9–11.

**TABLE 9:** Cluster 1 (from AbundanceBin on S7) partitioning obtained running MetaProb on it after unpaired reads reassignement.

Cluster	Species	Paired reads
Cluster 1.A	Species 1	4,370
	Species 2	15,285
	Species 3	3,211
	**Species 4**	**541,144**
	Species 5	22,428
Cluster 1.B	Species 1	4,077
	Species 2	812
	Species 3	3,649
	Species 4	37,410
	**Species 5**	**110,605**
Cluster 1.C	Species 1	51
	Species 2	7
	Species 3	21
	Species 4	3,967
	**Species 5**	**581,932**

The reads of the dominant species in the cluster have been highlighted in bold.

**TABLE 10:** Cluster 2 (from AbundanceBin on S7) partitioning obtained running MetaProb on it after unpaired reads reassignement.

Cluster	Species	Paired reads	Cluster	Species	Paired reads
Cluster 2.A	Species 1	4,698	Cluster 2.F	Species 1	554
	**Species 2**	**20,952**		**Species 2**	**30,002**
	Species 3	12,111		Species 3	0
	Species 4	3,449		Species 4	1
	Species 5	1,853		Species 5	0
Cluster 2.B	Species 1	6,508	Cluster 2.G	Species 1	133
	Species 2	64		Species 2	585
	**Species 3**	**6,628**		Species 3	5
	Species 4	757		Species 4	441
	Species 5	72		**Species 5**	**2,087**
Cluster 2.C	Species 1	2,381	Cluster 2.H	Species 1	1,608
	Species 2	258		**Species 2**	**3,111**
	**Species 3**	**91,188**		Species 3	299
	Species 4	53		Species 4	345
	Species 5	1		Species 5	3,039
Cluster 2.D	**Species 1**	**3,178**	Cluster 2.I	**Species 1**	**98,564**
	Species 2	1,246		Species 2	0
	Species 3	2,235		Species 3	1,079
	Species 4	347		Species 4	0
	Species 5	86		Species 5	0
Cluster 2.E	Species 1	268	Cluster 2.J	**Species 1**	**11,104**
	Species 2	766		Species 2	2
	**Species 3**	**1,257**		Species 3	774
	Species 4	114		Species 4	60
	Species 5	24		Species 5	41

The reads of the dominant species in the cluster have been highlighted in bold.

**TABLE 11:** Cluster 3 (from AbundanceBin on S7) partitioning obtained running MetaProb on it after unpaired reads reassignement.

Cluster	Species	Paired read
Cluster 3.A	**Species 1**	**3,281**
	Species 2	303
	Species 3	564
	Species 4	0
	Species 5	0
Cluster 3.B	Species 1	708
	Species 2	519
	**Species 3**	**1,502**
	Species 4	0
	Species 5	0
Cluster 3.C	Species 1	445
	Species 2	1,271
	**Species 3**	**1,660**
	Species 4	0
	Species 5	0

The reads of the dominant species in the cluster have been highlighted in bold.

##### 3.3.2.1 Analysis of Cluster 1

In ideal conditions, Cluster 1 generated by AbundanceBin should have contained only reads from the two most abundant species. However, as previously shown in Table 5, some noise from other species was also introduced. Nonetheless, the results confirm that MetaProb is able to effectively identify two sub-clusters covering about 90% of the reads of Cluster 1 and containing the majority of the reads from these two species: 92% of Cluster 1.A contains reads that belong to Species 4, while more than 99% of Cluster 1.C contains reads from Species 5. The remaining Cluster 1.B covers only 11% of the total reads of Cluster 1. Of these, 70% belong to Species 5, and 24% of reads belong to Species 4, with the remaining 6% consisting of reads from the minority species. The size and mixture of this cluster are, therefore, those of a “spurious” cluster possibly produced by the noise introduced by AbundanceBin, by a wrong estimate number of clusters from MetaProb, or by a combination of both.

It is important to highlight that MetaProb is naturally better suited to working with clusters that contain species with similar abundances. This is evident in the correct classification of Species 4 and 5. However, MetaProb encounters difficulties when the variation in species abundance is more marked, as in the case of Cluster 2. This aspect motivates the approach adopted in this experiment, which aimed to improve the performance of MetaProb by removing one of its weak points, a limitation similar to that found in other software based on DNA composition.

##### 3.3.2.2 Analysis of Cluster 2

Giving the reads of Cluster 2 as input to MetaProb caused the generation of 10 different clusters. Considering that 95% of the reads in Clusters 2 belong to Species 1, Species 2, and Species 3, this result was somehow unexpected. However, by carefully examining each cluster composition, we can observe that four of these clusters (2.A, 2.C, 2.F, and 2.I) contain more than 30,000 paired reads. Specifically, reads from Species 1 and Species 3 are mainly assigned, respectively, to Cluster 2.I and 2.C, and in both cases, they represent about 98% of the total composition of these clusters. Species 2 instead has been mainly split between Clusters 2.A and 2.F that together contain more than 90% of the reads of this species.

The remaining six clusters have been assigned a smaller number of reads and can either be seen as a relatively small erroneous splitting of a species (e.g., Cluster 2.J that is almost entirely composed of reads of Species 1, correct identification of a species that should not have been in Cluster 2 (Cluster 2.J mainly contains reads from Species 5), or a mixture generated by the intrinsic similarity in terms of k -mers that some species may share.

Overall, this in-depth analysis allows us to conclude that the output of MetaProb consists of four main clusters characterized by reads of the three species expected from this cluster, plus some noise. We will discuss later ideas on how to further improve this result.

##### 3.3.2.3 Analysis of Cluster 3

Once the unpaired reads had been moved, Cluster 3 contained only 10,253 pairs of paired-end reads, which represents less than 1% of the total number of reads in the dataset, confirming the fact that it is a “spurious” cluster generated by AbundanceBin. Although it exclusively contained reads from the three species with lower abundance, given their non-representative nature, MetaProb could not fully exploit its distinguishing power based on overlap and composition signature. Nonetheless, as can be seen in Table 11, it was able to clearly distinguish reads of Species 1 (80% of Cluster 3.A is composed of reads from this species), while the distinction between Species 2 and Species 3 was more difficult. It is worth noting that within Cluster 3, Species 1 has basically twice as many reads as the other two species. We speculate that this could have helped MetaProb in finding the overlaps it needs to build its initial clusters.

#### 3.3.3 Comparison with standalone tools

To summarize our results, we computed quality measures of the final binning obtained with those obtained by the single use of AbundanceBin and MetaProb. Moreover, we included the results of the currently available version of MetaCluster (5.0), which includes a low/high abundance partitioning phase before the final clustering.

Results shown in Table 12 support our intuition: among the datasets analyzed in previous studies, S7 was one of the most challenging as AbundanceBin struggles to obtain good precision, while MetaProb shows lower performances in both precision and recall with respect to other datasets. MetaCluster 5.0, which already includes a high/low abundance partitioning, showed the highest precision but the worst recall. Our combined approach slightly improves over MetaProb in terms of both precision (showing results comparable to those of MetaCluster) and recall, while it can maintain the high recall while doubling the precision with respect to AbundanceBin. The values of F-measure and ARI confirm the overall better performances of the proposed combined complementary approach.

**TABLE 12:** Standalone tools vs. MetaComBin combined framework on the dataset S7.

Dataset S7	MetaCluster	MetaProb	AbundanceBin	MetaComBin
Precision	**0.925**	0.818	0.477	0.912
Recall	0.671	0.745	**0.879**	0.812
F-measure	0.778	0.780	0.618	**0.859**
ARI	—	0.520	0.344	**0.730**

In bold the best performance for each metric.

### 3.4 Experimental analysis of dataset S9red

The analysis for S9red was performed similarly. We will present the results for this dataset in a more compact way.

#### 3.4.1 Step 1: clustering of reads based on abundances

We do not make any assumptions about the number of clusters when running the tools. Given S9red in input, AbundanceBin detected two major clusters rather than the expected three. After read pairing, we have Cluster 1 composed of 1,567,881 reads (96% of the total) and Cluster 2 composed of 65,646 reads. Cluster 2 is mainly composed (73%) of the two low-abundance species (Species 3 and Species 8), while Cluster 1 is composed of the great majority of reads of the medium- and high-abundance species. Note that high-abundance species are 1.5 more abundant than those with medium abundance. Because the difference between these two classes is not larger than 2, AbundanceBin is expected to struggle to distinguish between them, and, in fact, it puts them together in a single cluster.

#### 3.4.2 Step 2: partitioning of the abundance clusters

When applying MetaProb to Cluster 1, again without specifying the number of expected clusters k , we obtain many groups, specifically 21. However, eight such groups have a number of reads that is less than 1% of the size of the cluster and can be considered noise. If we focus on the nine groups that contain at least 5% of the reads in Cluster 1 (details in Table 13), we have that eight contain more than 99% of reads of the same species, and seven of those eight cover more than 70% of the reads of the same species (more than 88% for Species 1, 2, 5, 7, 9, and 10, and about 73% for Species 4). Species 6 is mainly split between two groups, covering about 60% and 26%, respectively. The remaining group, Cl7, shown in the table, covers most of the remaining reads of Species 4 (about 26%).

**TABLE 13:** Detailed partitioning of Cluster 1 (after unpaired-reads reassignment on the clusters obtained by AbundanceBin on S9red) with MetaProb. With %sp, we indicate the percentage of the majority species wrt the total number of reads for that species in the dataset (i.e., how well the group covers the majority species in it); with %sg, we indicate the percentage of the reads of the majority species wrt the total reads in that group (i.e., how well-defined the cluster is).

	Cl1	Cl5	Cl7	Cl8	Cl10	Cl11	Cl15	Cl19	Cl21
sp1	121	**79,102**	6	0	7	18	0	0	0
sp2	1,541	0	0	0	102	8	0	1,291	**198,707**
sp3	4,768	1,064	173	0	170	513	0	1	0
sp4	3,425	1,219	**121,953**	44	2	30	0	0	0
sp5	20,694	2,685	135	1	**141,016**	514	0	0	25
sp6	**46,579**	832	0	1	121	**109,026**	0	0	0
sp7	554	250	0	**175,985**	0	1	0	92	0
sp8	508	0	0	0	0	9	0	325	437
sp9	1,982	0	0	0	0	6	**266,944**	0	0
sp10	1,661	521	101	0	0	0	0	**211,589**	1,557
tot	81,833	85,673	122,368	176,031	141,418	110,125	266,944	213,298	200,726
%sp	25.65	96.45	72.91	96.39	79.36	60.05	95.64	88.85	91.61
%sc	56.92	92.33	99.66	99.97	99.72	99.00	100.00	99.20	98.99
id	Species 6	Species 1	Species 4	Species 7	Species 5	Species 6	Species 9	Species 10	Species 2

The reads of the dominant species in the cluster have been highlighted in bold.

Among the remaining groups with a size between 1% and 5% of Cluster 1 (not shown in the table because of space constraints), we have one well-defined group (about 86% of reads in it) covering 44% of Species 8, another well-defined group with 33% of the reads of Species 3, and a less well-defined group mostly composed by residual reads of Species 10.

When applying MetaProb to Cluster 2, we obtain five groups. Two of them are composed of 90% of reads from Species 8, covering together 39% of the reads of the species. The other two well-defined groups cover about 42% of Species 2. The remaining group is less well defined. The majority species in it, Species 10, covers 44% of the group. However, considering that Cluster 2 is much smaller than Cluster 1, these results can be considered noise as they represent only 2% of the reads of that species, which is much better represented by the groups Cl19 obtained in Cluster 1.

To summarize our finding, after applying our pipeline, we were able to identify the high- and medium-abundance species with both very high precision and recall. The reads of the low-abundance species were split between the two clusters found by AbundanceBin (57%–43% For species 3 and 54%–46% for Species 8). It is possible that the phylogenetic closeness of some species made their reads difficult to partition based on abundance only. However, when applying the second step of our analysis, the reads of these species were well separated from the others.

#### 3.4.3 Comparison with standalone tools

Table 14 shows the result of the comparison of our pipeline with respect to those of the two tools run separately. Unfortunately, we were not able to run MetaCluster on our computer system. Because we needed to reduce S9 to be able to run AbundanceBin, we could not refer to MetaCluster to results appearing in previous papers, as we did for the analysis of S7.

**TABLE 14:** Standalone tools vs. MetaComBin combined framework on the reduced dataset S9.

Dataset S9red	MetaProb	AbundanceBin	MetaComBin
Precision	**0.921**	0.191	**0.921**
Recall	0.785	**0.901**	0.824
F-measure	0.843	0.315	**0.870**
ARI	0.778	0.014	**0.833**

In bold the best performance for each metric.

### 3.5 Results on the synthetic dataset MIXK

The results of the application of our pipeline to the synthetic dataset MIXK are shown in Table 15 and in Table 16 for each of the two clusters, Cluster 1 and Cluster 2, detected by AbundanceBin and then partitioned by MetaProb. For ease of visualization, we report only the cluster with a size larger than 2% of the dataset.

**TABLE 15:** Detailed partitioning of Cluster 1 (after unpaired-reads reassignment on the clusters obtained by AbundanceBin on MIXK) with MetaProb. With %sp, we indicate the percentage of the majority species wrt the total number of reads for that species in the dataset (i.e., how well the group covers the majority species in it); with %sg, we indicate the percentage of the reads of the majority species wrt the total reads in that group (i.e., how well-defined the cluster is). Clusters with a size greater than 2% of the total size of the dataset are reported.

	cl1.1	cl1.2	cl1.3	cl1.4	cl1.5	cl1.6	cl1.7	cl1.8
sp1	1,584	1,950	325	556	0	0	0	418
sp2	2	0	0	0	154	264	43	1
sp3	8,260	16,298	14,599	16,913	5	0	0	552
sp4	935	296	260	276	35,558	2,368	1,879	284
sp5	18	3	0	0	3,570	28,023	4,168	27
sp6	35	50	7	0	1,723	1,991	59,940	230
sp7	23,071	8,228	5,497	9,807	91	36	4	2,280
sp8	6,354	8,284	1,835	6,014	82	74	1	17,831
sp9	1,593	812	229	144	25	29	0	317
sp10	5,504	2,266	27,820	6,600	0	0	0	7
tot	23,071	16,298	27,820	16,913	35,558	28,023	59,940	17,831
sp	43.84	27.27	46.02	28.29	68.69	51.65	61.34	31.76
sc	48.72	42.68	55.01	41.96	86.29	85.48	90.77	81.25
id	Species 7	Species 3.b	Species 10.a	Species 3.a	Species 4	Species 5	Species 6	Species 8

**TABLE 16:** Detailed partitioning of Cluster 2 (after unpaired-reads reassignment on the clusters obtained by AbundanceBin on MIXK) with MetaProb. With %sp, we indicate the percentage of the majority species wrt the total number of reads for that species in the dataset (i.e., how well the group covers the majority species in it); with %sg, we indicate the percentage of the reads of the majority species wrt the total reads in that group (i.e., how well-defined the cluster is). Clusters with a size greater than 2% of the total size of the dataset are reported.

	cl2.1	cl2.2	cl2.3	cl2.4
sp1	19,324	60,938	5,663	0
sp2	2,292	152	103	288,161
sp3	265	1,179	1,241	1
sp4	556	50	12	30
sp5	55	4	0	39
sp6	747	96	5	227
sp7	289	437	376	0
sp8	827	860	274	2
sp9	126,308	21,444	7,717	16
sp10	652	2,181	15,355	0
tot	151,315	87,341	30,746	288,476
sp	75.31	66.58	25.40	93.55
sc	83.47	69.77	49.94	99.89
id	Species 9	Species 1	Species 10.b	Species 2

It is interesting to note that among the bigger clusters, it is possible to associate a distinct species to each cluster, except for Species 3 and Species 10, which were split between two clusters.

Finally, we report the comparison between AbundanceBin and MetaProb, which are run separately and as a pipeline, in Table 17. Even for this dataset, the combined approach showed generally better behavior than the tools run separately.

**TABLE 17:** Standalone tools vs. the MetaComBin combined framework on the synthetic dataset MIXK.

Dataset MIXK	MetaProb	AbundanceBin	MetaComBin
Precision	0.657	0.377	**0.787**
Recall	0.654	**0.928**	0.684
F-measure	0.656	0.536	**0.732**
ARI	0.458	0.253	**0.740**

In bold the best performance for each metric.

## 4 Conclusion and future work

This study explored the combined use of complementary tools for metagenomics read binning in order to improve the overall quality of the binning process when some species have the same abundance ratio, and no knowledge of the actual number of species is given, as in a realistic context. Our results on three datasets that were difficult to analyze with other popular read-binning tools showed that a combined framework could exploit the strengths of different read-binning approaches to obtain better values in terms of clustering quality metrics than a single tool. Moreover, although we did our test with two specific metagenomic read-binning approaches based on abundance and overlaps (AbundanceBin and MetaProb), in principle, our framework can potentially combine any two tools with these characteristics.

Our analysis also pointed out some aspects that can be the subject of future studies: for example, the total number of clusters produced by the current pipeline is larger than the exact number of clusters. Although some over-estimation is expected because the exact estimation of the number of clusters is a challenge itself, and the problem is common to all the tools when k is not given in input, it would be interesting to see if and to which extent this estimation can be improved. Two directions we plan to investigate aim at: i) the reduction of the noise introduced by AbundanceBin not taking into consideration paired-end reads by developing a strategy that clusters paired-end reads together or by adopting other strategies for reads reassignment, and ii) the further merge of “minor” clusters by adding a post-processing step to our pipeline.

## Data Availability

The original contributions presented in the study are included in the article/supplementary material; further inquiries can be directed to the corresponding author.

## Footnotes

1 A preliminary version was presented at ICCABS2023.

2
https://bitbucket.org/samu661/metaprob/src/master/
